# Chromosomal Abnormality in Men with Impaired Spermatogenesis

**Published:** 2014-03-09

**Authors:** Dana Mierla, Dumitru Jardan, Veronica Stoian

**Affiliations:** 1 Life Memorial Hospital, Bucharest, Romania; 2 Department of Genetics, Faculty of Biology, University of Bucharest, Bucharest, Romania

**Keywords:** Chromosomal Abnormality, Chromosome Microdeletion, Male Infertility, Azoospermia, Oligozoospermia

## Abstract

Background: Chromosomal abnormalities and Y chromosome microdeletions are regarded as two most frequent genetic causes associated with failure of spermatogenesis in the Caucasian population.

Materials and Methods: To investigate the distribution of genetic defects in the Romanian population with azoospermia or severe oligozoospermia, karyotype analysis by G-banding was carried out in 850 idiopathic infertile men and in 49 fertile men with one or more children. Screening for microdeletions in the azoospermia factor (AZF) region of Y chromosome was performed by multiplex polymerase chain reaction (PCR) on a group of 67 patients with no detectable chromosomal abnormality. The results of the two groups were compared by a two-tailed Fisher’s exact test.

Results: In our study chromosomal abnormalities were observed in 12.70% and 8.16% of infertile and fertile individuals respectively.

Conclusion: Our data suggests that infertile men with severe azoospermia have higher incidences of genetic defects than fertile men and also patients from any other group. Infertile men with normal sperm present a higher rate of polymorphic variants. It is important to know whether there is a genetic cause of male infertility before patients are subjected to intracytoplasmic sperm injection (ICSI) or testicular sperm extraction (TESE)/ICSI treatment.

## Introduction

Male infertility is a common and severe health problem affecting 7% of populations (1). Infertility not only affects one’s ability to have children, but also has emotional, psychological, familial and social effects. Despite the prevalence and significance of this health problem, resources and attention have not been sufficiently focused on this important issue. The most common causes of male infertility are abnormal sperm delivery, chromosomal abnormalities, defective pituitary gland function, infections of the male accessory glands and overexposure to certain environmental factors (2).

Most men presenting with infertility are found to have idiopathic oligo-astheno-terato- zoospermia (OAT) (3). The etiology of male infertility is unknown in approximately one third of the patients. The unaccountable forms of male infertility may be caused by several fac- tors, such as chronic stress, endocrine disruption due to environmental pollution and genetic abnormalities (4-6). There is genetic predisposition to these pathologies such as varicocele (increased scrotal temperature) (1). Also, environmental factors are implicated in the onset and progression of male infertility (7). 

Of the 7% of men suffering from infertility, 40% have idiopathic infertility. The cause of infertility in these men seems to be due to underlying genetic abnormalities (1). Chromosomal abnormalities are one of the major causes of human infertility as they interfere with spermatogenesis. Study of human chromosomes plays a key role in diagnosis, prognosis and monitoring of chromosomal abnormalities.

 In infertile males, abnormal karyotype is more frequent than in the general population (8). Approximately 8 to 15% of men diagnosed with oligo or *azoospermia* present microdeletion on the long arm of chromosome Y, which by loss of specific DNA segments, vital genes needed for sperm production may be lost (9).

Male factor problems commonly manifest through an alteration of one or more semen parameters and the following terminology are used to describe these changes:

*Azoospermia*–spermatozoa cannot be found in a semen sample.
*Aspermia*–no semen sample was ejaculated.
*Necrozoospermia*–all spermatozoa in the semen sample are dead.
*Globozoospermia*–none of the spermatozoa have an acrosomal cap.
*Oligozoospermia*–the concentration of spermatozoa is less than 20 million/ml of the ejaculate.
*Asthenozoospermia*–less than 50% of spermatozoa in the semen sample exhibit forward motility.
*Teratozoospermia*–the proportion of spermatozoa with normal shape is less than 30% (10).

At least 15 gene families, that are involved in spermatogenesis, are found on the long arm of chromosome Y (Yq) (11-14). Among infertile patients with *azoospermia* and oligozoospermia, Y chromosome microdeletions have been identified as causal factors. Y chromosome microdeletions are grouped in three main regions (AZFa, AZFb and AZFc) on the Yq arm (15).

## Materials and Methods

From 2007 to April 2011, 850 infertile men (including 350 patients with oligo-asthenoteratozoospermia, 150 patients with severe oligozoospermia, 100 patients with *azoospermia*, 100 patients with teratozoospermia and 150 with normal semen analysis) who were referred to the Department of Reproductive Medicine at the Life Memorial Hospital of Bucharest in Romania were investigated for this retrospective study. 49 normozoospermic male donors with normal semen parameters (sperm count >20×10^6^ /ml, progressive motility >50% and normal morphology >30%) and proven fertility (with one or more children) were included as controls. All patients were initially evaluated by a andrologist and conventional diagnostic work-up including patient’s history, genital examination, ultrasonography and hormone analyses were performed. None of them had any history of childhood disease, environmental exposure, radiation exposure or prescription drug usage that could account for their infertility. The median age of patients was 35.4 years (range 26-50 years).

Patients had been referred for chromosomal analysis, fluorescence in situ hybridization (FISH) for detection of Yp chromosome microdeletions and polymerase chain reaction (PCR) for 15 sites of AZF region on Y chromosome. In this prospective study we investigated infertile men prior to intracytoplasmic sperm injection (ICSI) treatment. Informed consent was obtained from the patients and controls prior to collection of heparinized blood samples.

Karyotype analysis was performed on peripheral blood lymphocytes. After 72 hours culture, the cells were harvested, hipotonised and fixed using 3:1 methanol: acetic acid. The metaphases were spread on slides. At least 10 metaphases were analyzed for each case and chromosomal abnormalities were reported according to the recommendations of the International System for Chromosome Nomenclature (ISCN 2009) (16, 17).

FISH was performed for 629 cases (using commercially available Vysis (Abbott) FISH probes which are complementary to the region of interest on a particular chromosome) (18).

PCR was performed to screen the microdeletions in the AZF region of the Y chromosome. Genomic DNA was extracted according to standard procedure from peripheral blood samples (19). Each patient was analysed for the presence of sequence tagged sites (STS) in the AZFa, AZFb and AZFc regions (Table 1) (20). The STS probes used were sY84 and sY86 (AZFa), sY127 and sY134 (AZFb), sY254 and sY255 (AZFc), and SRY and ZFX/ZFY (controls).

This original study was approved by The Ethics Committee of Life Memorial Hospital Statistical Analysis: The results of the two groups were compared by a two-tailed Fisher’s exact test and calculated online using Graph Pad (http://www.graphpad.com/quickcalcs/contingency1.cfm8). 

**Table 1:** Primer sequences of the sequence-tagged-sites (STSs) used in the detection of AZF loci (AZFa, AZFb and AZFc) and SRY


STS	Sequence 5' - 3'	Locus	Size (bp)

**sY86-F**	GTG ACA CAC AGA CTA TGC TTC	AZFa	320
**sY86-R**	ACA CAC AGA GGG ACA ACC CT		
**sY127-F**	GGC TCA CAA ACG AAA AGA AA	AZFb	274
**sY127-R**	CTG CAG GCA GTA ATA AGG GA		
**sY254-F**	GGG TGT TAC CAG AAG GCA AA	AZFc	400
**sY254-R**	GAA CCG TAT CTA CCA AAG CAG C		
**sY14-F**	GAA TAT TCC CGC TCT CCG GA	SRY	495
**sY14-R**	GCT GGT GCT CCA TTC TTG AG		
**sY84-F**	AGA AGG GTC TGA AAG CAG GT	AZFa	326
**sY84-R**	GCC TAC TAC CTG GAG GCT TC		
**sY134-F**	GTC TGC CTC ACC ATA AAA CG	AZFb	301
**sY134-R**	ACC ACT GCC AAA ACT TTC AA		
**sY255-F**	GTT ACA GGA TTC GGC GTG AT	AZFc	126
**sY255-R**	CTC GTC ATG TGC AGC CAC		
**sY14-F**	GAA TAT TCC CGC TCT CCG GA	SRY	495
**sY14-R**	GCT GGT GCT CCA TTC TTG AG		


## Results

**Fig 1:**
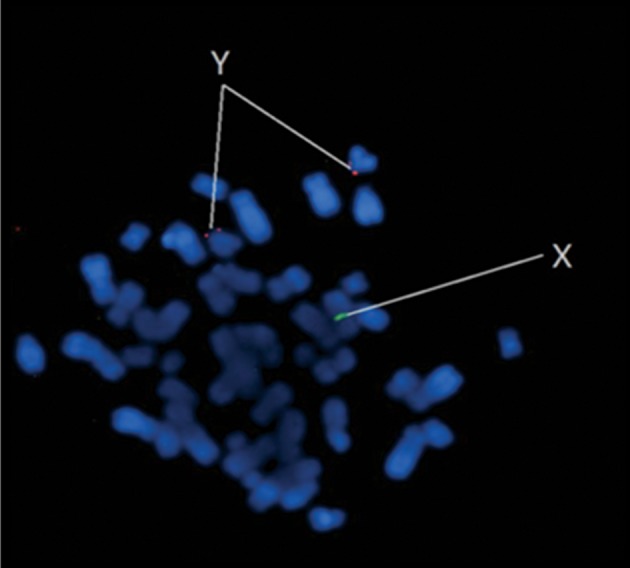
Isochomosom Y. Specific pattern of signals: an X chromosome specific signal (CEPX) and two signals characteristic of Y chromosome (SRY LSI). Result: 47,XYY. ish(CEPXx1) (SRYx2).

Karyotyping was carried out in 850 infertile men with impaired spermatogenesis. As shown in table 2, a total of 108 patients (12.70%) had chromosomal abnormalities: 6 azoospermic patients with Klinefelter’s syndrome (47,XXY), 2 patients with 47,XYY syndrome (confirmed by FISH) (Fig 1) and 100 patients with structural chromosomal abnormality [of which 77 patients (71.3%) had polymorphic variants]. Only 2 out of 67 patients tested (3%) exhibited deletions on the long arm of Y chromosome, one of them being azoospermic and another oligozoospermic. In the control group we found 4 individuals with polymorphic variants: one case with an inversion on chromosome 9, two cases with chromosome heteromorphisms qh+ and one with a fragile site on chromosome 21 (Table 3). Types of chromosomal aberrations detected in men with infertility problems are presented in table 4. The most common chromosomal variants observed in infertile men are chromosomal polymorphisms (71.30%) (Table 5).

**Table 2:** Chromosomal abnormalities in infertile men


Normal karyotype	Infertile maleNo./%	Fertile maleNo./%

**CA**	108(12.70% )	4(8.16%)
**Numerical CA**	8(7.41%)	-
**Structural CA**	100(92.59%)	4(8.16%)


Screening of AZF microdeletions was carried out in the 67 patients including 46 infertile patients with *azoospermia* and 21 infertile patients with severe oligozoospermia with normal karyotype. In our study 2 of the 67 patients tested (3%) showed a Yq11 microdeletion, involving the AZFc locus, 1 of them being azospermic and 1 oligospermic and no patients presented microdeletions on Yp chromosome (out of 504 patients). No deletion in AZF region was found in fertile controls.

**Table 3:** Chromosomal abnormality in studied groups


Patients	Autosomal abnormalities	Sex abnormalities	Polymorphic variants	Total abnormalities (autosomal + sex chromosome)	Total

**Infertile male (n=850)**	20(2.35%)	11(1,29%)	77(9.06%)	31(3.65)	108(12.71%)
**OAT (n=350)**	5(1,43%)	1(0.29%)	10(2.86%)	6 (1.71%)	16(4.57%)
**Azoospermia (n=100)**	8(8%)	7(7%)	15(15%)	15 (15%)	30(30%)
**Oligozoospermia (n=150)**	2(1.33%)	1(0.67%)	5(3.33%)	3 (2%)	8(5.33%)
**Teratozoospermia (n=100)**	1(1%)	-	11(11%)	1 (1%)	12(12 %)
**Normal semen analysis(n=150)**	4(2.67%)	2(1.33%)	36(24%)	6 (4%)	42(28%)
**Fertile male (n=49)**	-	-	4(8.16%)	-	4(8.16%)


**Table 4:** Numerical and structural abnormalities in infertile men


Chromosomal aberrations		Karyotype	No. of cases	Frequency %

**Structural abnormalities**	Inversion	46,XY,inv(9)(p11q13)	22	2.59%
		46,XY,inv(9)(p11q12)	3	0.35%
		46,XY,inv(3)(p11q11.2)inv(9)(p11q13)	1	0.12%
		46,XY,inv(1)(q23p13)	1	0.12%
		46,XY,inv(1)(q13p31)	1	0.12%
		46,XY,inv(10)(p11.2q21)	1	0.12%
		46,XY,inv(5)(pterq13)	1	0.12%
	Deletion	46,X,delY(q11.2)	1	0.12%
		46,X,delY(q12)	1	0.12%
	**Translocation**	46,XY,t(1;19)(p13;13.3)	1	0.12%
		46,XY,t(3;13)(p21;p11.2)	1	0.12%
		46,XY,t(1;9)(q11;p13)	2	0.24%
		45,XY,t(13;14)(q10q10)	5	0.59%
		45,XY,t(14;15)(q10q10)	1	0.12%
		46,XY,t(9;10)(q12q26)	1	0.12%
		46,XY,t(9;3)(q32q28)	1	0.12%
		46,XY,t(1;4)(q43q13)	1	0.12%
		46,XY,t(7;8)(q31.1q24)	1	0.12%
		46,XY,t(3;6)(q28;q13)	1	0.12%
**Numerical abnormalities**	Klinefelter SyndromSyndrom XX	47,XXY	6	0.71%
		47,XYY	2	0.24%
		46,XX	1	0.12%


**Table 5:** Chromosomal polymorphisms in infertile men


Chromosomal polymorphic variations	Karyotype	No. of cases	Frequency %

**Heteromorphisms qh+fragile sites**	46,XY,1qh+	10	9.26%
	46,XY, 9qh+	9	8.33%
	46,XY,16qh+	2	1.85%
	46,X Yqh+	3	2.78%
	46,XY, fra(17)	7	6.48%
	46,XY, fra(16)	4	3.70%
**pseudo satellites**	46,XY, 14ps+	5	4.63%
	46,XY, 15ps+	2	1.85%
	46,XY, 21ps+	2	1.85%
	46,XY, 22ps+	8	7.41%


## Discussion

This study was designed to explore the implication of chromosomal abnormalities (CA) in male infertility. In our study constitutional chromosomal abnormalities were identified in 12.70% (71.3% polymorphic variants) of infertile patients and 8.16% (100% polymorphic variants) in the control group. Structural chromosomal abnormalities were present in a high proportion of men with infertility problems. In fertile controls, structural abnormality was detected in 4 cases only and no deletion in AZF region was found.

We identified 6 cases of Klinefelter’ syndrome, which is reported to be the most frequent chromosomal aberration causing *azoospermia* in men followed by Yq deletions (21). The prevalence of Klinefelter’ syndrome among infertile men is very high, up to 5% in severe oligozoospermia and 10% in *azoospermia* (22). We identified Klinefelter’ syndrome in 6% of patients with *azoospermia* but it was absent in all other groups. The incidence of sex chromosome aneuploidies was statistically significantly different for *azoospermia* group versus any other group (7% vs. at most 1.33%, p=0.018).

The incidence of chromosomal abnormalities in patients with *azoospermia* was significantly higher than that in patients of all other groups (15% vs. at most 4% for patients with normal semen) which was similar to other studies (21, 23). There was no significant difference in the incidence of autosomal abnormalities between infertile patients with OAT (1.43%), oligozoospermia (1.33%) and teratozoospermia (1%).

We identified a very high prevalence of polymorphic variants in infertile men with normal spermogram (24%). This prevalence is statistically higher than for any other group (24% vs. at most 15%, p=0.001). This might suggest a role for polymorphic variants in human fertility.

The most common reported clinical diagnosis among patients with inversion of chromosome 9 is *azoospermia* (24). This finding is similar to the present study. Pericentric inversion of chromosome 9, inv(9) (p11q12)/inv(9) (p11q13) is a common chromosomal rearrangement and some cytogeneticists consider it as a normal variant, generally without phenotypic effects (25). In this study, the inversion of chromosome 9 was found in 23.15% of patients with chromosomal abnormalities. Other chromosomal variants with a high incidence were 1qh + (9.26%), 9 qh + (8.33%) and 22ps + (7.41%). The least common polymorphic variation found in infertile couples usually occured in the paracentric heterochromatin on the long arms of chromosomes 16 (16qh + 1.85%), the short-arm regions of D and G group chromosomes (15ps + 1.85% and 21ps + 1.85%), and the distal heterochromatin of the Y chromosome (Yqh + 2.78%). In addition to Y chromosomal heteromorphism, other heteromorphisms related to infertility have also been described: heteromorphisms shown by short-arm regions of D and G group chromosomes and heteromorphisms shown by paracentric long-arm regions of chromosomes 1, 9 and 16, and inv(9) (26-28).

The exact mechanism by which chromosomal abnormalities induce infertility is still not completely understood. Some authors suggest that presence of abnormal chromatin interferes with meiotic division and affects sperm production (29). Genetic abnormalities which affect spermatogenesis may cause abnormal embryonic development, which in turn can lead to recurrent miscarriages (24, 30, 31). The infertile patients with normal karyotype, were further investigated for the presence of microdeletions on the short arm of chromosome Y and in the three AZF loci on Yq11. *azoospermia*, severe oligozoospermia and oligozoospermia are characterized by complete absence of sperm in semen, a sperm cell count <5×10^6^ cells/ml, and a sperm cell count >5×10^6^ and <20×10^6^ cells/ml respectively. Microdeletions on the short arm of chromosome Y and in the three AZF loci on Yq11 cause severe testiculopathies and infertility in 2% of cases (12, 32-35). The prevalence of Yq microdeletions increases to 15-20% of cases in males affected by severe oligozoospermia or non-obstructive *azoospermia* 9 (15, 35-38). We found no significant difference in the frequencies of AZF microdeletions between patients with *azoospermia* and those with severe oligozoospermia. Only 2 infertile men were diagnosed with microdeletions of the AZFc region. No microdeletions were detected in either AZFa, AZFb or AZFd. In one male subject we found a 46,XX karyotype (FISH analysis revealed an SRY signal on the short arm of the chromosome X (XX male syndrome) and only 2 males who presented karyotype 46,X,delY(q11.2) on cytogenetic analysis.

Infertile men have a higher risk of constitutional chromosomal rearrangements which may be the cause of infertility (39).

However, because of the limited size of the control group in the present study, the actual frequency of chromosome abnormalities needs to be investigated in a further study with a larger control group.

## Conclusion

The incidence of chromosome abnormalities in patients with *azoospermia* is higher than in any other groups both when autosomal aberrations or sex chromosome aneuploidies are compared. Klinefelter’ syndrome was the most frequent chromosomal aberration in azoospermic men. This association has been previously reported as such.

On the other hand, normospermic men carried the most frequent aberration (polymorphic variants) which was 1.6 times more frequent than in azoospermic men and 3 times more frequent than in fertile men.

The high rate of chromosomal abnormalities among infertile men strongly suggests the need for cytogenetic analysis and detection of Y chromosome microdeletions prior to the application of assisted reproduction techniques.
